# Two new species of the genus *Formosatettix* Tinkham, 1937 (Orthoptera, Tetrigidae) from Guizhou and Chongqing, PR China

**DOI:** 10.3897/zookeys.936.49552

**Published:** 2020-05-28

**Authors:** Ling-Sheng Zha, Xiao-Min Wu, Jian-Hua Ding

**Affiliations:** 1 School of Life Sciences, Huaibei Normal University; Huaibei 235000, China Huaibei Normal University Huaibei China

**Keywords:** ecology, habit, Karst Region, taxonomy, Tetriginae, Tetrigoidea

## Abstract

Two new pygmy grasshopper species are described from PR China and are assigned to *Formosatettix* Tinkham, 1937, a large Asian tetrigin genus composed of species with reduced tegmina and hind wings: *F. leigongshanensis* Zha & Ding, **sp. nov.** from Guizhou and *F. wulongensis* Zha & Ding, **sp. nov.** from Chongqing. We provide descriptions of morphology and habit, supplemented with photographs. Flying organs of the genus *Formosatettix* are discussed and the genus is compared with other Asian genera with reduced flying organs, such as *Formosatettixoides* Zheng, 1994 and *Alulatettix* Liang, 1993 in Tetriginae, *Deltonotus* Hancock, 1904, *Epitettix* Hancock, 1907 and *Pseudepitettix* Zheng, 1995 in Cladonotinae, and *Macromotettixoides* Zheng, Wei & Jiang, 2005 and *Pseudomacromotettix* Zheng, Li & Lin, 2012 in Metrodorinae.

## Introduction

The genus *Formosatettix* Tinkham, 1937 (subfamily Tetriginae) was originally established for only two species from Taiwan, China – *F. arisanensis* Tinkham, 1937 (type species) and *F. karenkoensis* Tinkham, 1937, but today it is a large genus composed of 68 known species in China, Japan, Korea, Nepal, Pakistan and Russia (Wei et al. 2019, Cigliano et al. 2020). *Formosatettix* is similar to the Tetriginae genera *Alulatettix* Liang, 1993 and *Formosatettixoides* Zheng, 1994. Members of *Alulatettix* have a pair of conspicuous tegminal sinus (Zhang et al. 2014), while those of *Formosatettixoides* have visible tegmina and hind wings (Zha and Zheng 2014). Member of *Formosatettix*, on the other hand, lack a pair of tegminal sinus and both tegmina and hind wings are invisible (Wei et al. 2019). *Formosatettix* is also similar to the Cladonotinae genera *Deltonotus* Hancock, 1904, *Epitettix* Hancock, 1907, and *Pseudepitettix* Zheng, 1995 [which is a likely synonym of *Epitettix* as suggested by Deng (2016) and Zha et al. (2017b)], and to the Metrodorinae genera *Macromotettixoides* Zheng, Wei & Jiang, 2005 and *Pseudomacromotettix* Zheng, Li & Lin, 2012 [syn. of *Macromotettixoides* as suggested by Zha et al. (2017a)]. Relations among the aforementioned genera have never been systematically investigated.

During investigations of pygmy grasshoppers in PR China, we have collected new data on members of the genus *Formosatettix*. In this study, we introduce two new members of the genus *Formosatettix*, namely *F. leigongshanensis* Zha & Ding, sp. nov. and *F. wulongensis* Zha & Ding, sp. nov., from Southwest China. At the same time, we provide brief discussion on the morphology of the flying organs of *Formosatettix*, and compare the genus to allied genera.

## Material and methods

**Photography.** Specimens were photographed using Canon EOS 800D with 100 mm macro lens, and partial images were stacked using Photoshop CS6. Photographs of the habitat were made using Nikon Coolpix P520.

**Terminology.** Morphological terminology and measurements follow Zheng (2005), Tumbrinck (2014) and Muhammad et al. (2018). Measurements are given in millimeters (mm).

**Depository.** Type and voucher specimens are deposited in the Specimen Room of the School of Life Sciences, Huaibei Normal University (HNU), Huaibei, Anhui Province, China.

**Taxonomy.** Taxonomy follows Cigliano et al. (2020) (= Orthoptera Species File).

## Taxonomy

### *Formosatettix* Tinkham, 1937

#### Formosatettix leigongshanensis

Taxon classificationAnimaliaOrthopteraTetrigidae

Zha & Ding sp. nov.

BA2753BD-59C5-52F9-830D-97FDB9C80026

http://zoobank.org/894716AD-59D6-40D5-8283-C5737BD64A21

Figs 1 , 2

##### Diagnosis.

*Formosatettix serrifemora* Deng, 2019 was reported from Liupanshui (Yushe) and Suiyang (Kuankuoshui), Guizhou, China (Wei et al. 2019), and is geographically closest to our new species. We have collected the species (9♂20♀) from the Tongzi County (Baiqing Natural Reserve), also in Guizhou. Apart from the number of antennal segments (14 or 15) and presented tegmina and hind wings (closely similar to *F. leigongshanensis* sp. nov.), our collections are identical to the description and photographs of *F. serrifemora* (14-segmented, tegmenulum and hind wing absent; Wei et al. 2019).

*Formosatettix leigongshanensis* sp. nov. is similar to *F. serrifemora*, but the latter has a narrower scutellum, an acutely angled anterior margin of the pronotum in dorsal view, undulate ventral margins of the fore and mid femora, and undulate dorsal margin of the hind femur (Wei et al. 2019, fig. 7b). The main differences between the two species are outlined in Table 1.

**Table 1.** Main differences between *Formosatettix serrifemora*, *F. leigongshanensis* sp. nov. and *F. wulongensis* sp. nov.

	***F. serrifemora***	***F. leigongshanensis* sp. nov.**	***F. wulongensis* sp. nov.**
Anterior margin of the vertex	Elevated, arcuate, strongly surpassing the anterior margin of the compound eyes	Elevated, arcuate, strongly surpassing the anterior margin of the compound eyes	Low, straight, slightly surpassing the anterior margin of the compound eyes
Antennae	14–15 segments (♂, ♀), mid segments 2.5–3.0× as long as wide	15(♂)–16(♀) segments, mid segments 4–5× as long as wide	15(♂)–16(♀) segments, mid segments 4–5× as long as wide
Scutellum between the antennal grooves	Visibly narrower than diameter of scapus	Visibly wider than diameter of scapus	Visibly wider than diameter of scapus
Anterior margin of the pronotum	Acutely angled	Obtusely angled	Acutely angled
Median carina of the pronotum in lateral view	Low and arcuate	Low and arcuate	High and arcuate
Apex of hind pronotal process from dorsal view	Pointed-rounded	Pointed-rounded	Broadly arcuate
Ventral margins of fore and mid femora	Teeth present	Straight	Teeth present
Dorsal margin of the hind femur before the antegenicular tooth	Three teeth present clearly	Teeth absent	Three teeth present clearly

*Formosatettix leigongshanensis* sp. nov. is also similar to *F. changbaishanensis*Yuan et al., 2006 from Jilin, *F. yunnanensis* Zheng, 1992 from Yunnan, China, and *F. martensi* Ingrisch, 2001 from Nepal (Panchthar). Some of the main diagnostic differences are that in *F. changbaishanensis*: 1) vertex is 1.67♂–1.76♀ times as wide as one eye; 2) middle segments of the antennae are 5.16♀–6.3♂ times as long as wide; 3) prozonal carinae are contracted backwards; and 4) apex of the posterior angle of the lateral lobe is rounded (Yuan et al. 2006); in *F. yunnanensis*: 1) facial carinae before the eyes are indistinctly concave; 2) antennae are inserted slightly above the lower margin of the eyes; 3) scutellum is as wide as the diameter of scapus; 4) ventral margins of the fore and mid femora are undulate; and 5) apex of the posterior angle of the lateral lobe is rounded (Zheng 1992); and in *F. martensi*: 1) face is distinctly inclined and the frontal costa together with the medial carina of the vertex forms an acute angle; 2) anterior margin of the pronotum is slightly projected forwards and only reaches the posterior margin of the eyes; 3) ventral margin of the mid femur is undulate; and 4) the area between the internal and external lateral carinae of the pronotum is much narrower (Ingrisch 2001, Cigliano et al. 2020).

The new species is the second *Formosatettix* species, after *F. serrifemora*, reported in Guizhou Province, China.

**Figure 1.**
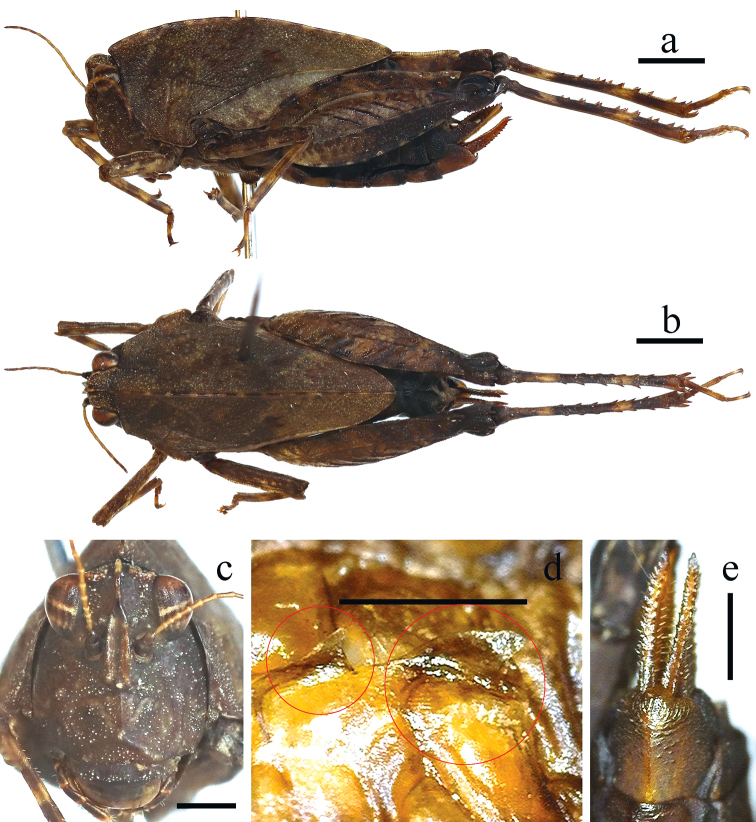
Female of *Formosatettix leigongshanensis* sp. nov. **a** body in lateral **b** body in dorsal view **c** head in frontal view **d** wings in lateral view (tegmenulum in the smaller circle, hind wing in the bigger circle) **e** subgenital plate in ventral view. Pictures **a, b** were stacked using Photoshop CS6. Scale bars: 2 mm (**a, b**), 1 mm (**c–e**).

**Figure 2.**
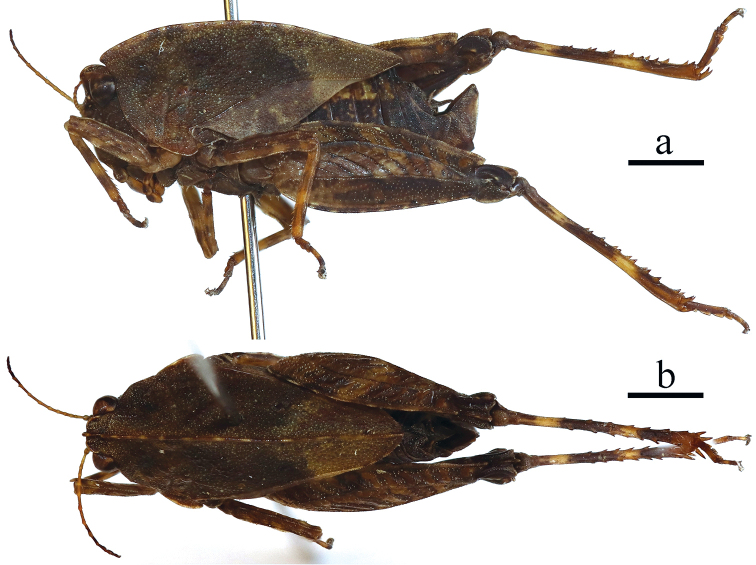
Male of *Formosatettix leigongshanensis* sp. nov. **a** body in lateral view **b** body in dorsal view. Pictures were stacked using Photoshop CS6. Scale bars: 2 mm.

##### Detailed description of females.

***General appearance*.** Body stout and short, size moderate, surface smooth and covered with numerous fine granules.

***Head*.** Vertex slightly below the anterior margin of the pronotum, slightly roof-like, anterior part higher than posterior part and center part higher than both sides, 2 times as wide as a compound eye; anterior margin somewhat arcuate, clearly surpasses the anterior margin of eyes; lateral carinae distinct, folded upwards and slightly over the top of eyes; medial carina distinct and compresso-elevated in anterior half, almost touching the median carina of the pronotum; paired fossulae distinct, rounded. In lateral view face nearly vertical, frontal costa together with medial carina rounded; facial carinae above superior ocelli concave, between antennal grooves arcuate forwards. In frontal view frontal costa bifurcates into facial carinae at the lower one-third of between anterior margin of vertex and upper margin of superior ocelli, and run nearly parallel downwards; scutellum deep and wide, between grooves 1.3–1.4 times as wide as the diameter of scapus. Eyes globose and elevated over the anterior margin of the pronotum, but clearly lower than top of vertex; superior ocelli placed at the level of lower one-third of the inner margins of eyes. ***Antenna*.** Antenna filiform, 16-segmented, inserted slightly below the lower margin of eyes, with 9–11^th^ segments longest and 4–5 times as long as wide.

***Pronotum*.** Pronotum distinctly compresso-elevated, surface smooth, between sulci somewhat swollen at the base of median carina and a little concave on both sides of the discus. The anterior margin projected forwards and reaching the middle of eyes, in dorsal view obtusely angled; prozonal carinae extend to the anterior sulcus, parallel, indistinct; hind pronotal process short, only reaching 3/4 of hind femur, apex pointed-rounded. Median carina of pronotum lamellate, in lateral view low arcuate; lower margin of hind process curved, internal lateral carina slightly incurved, the area between internal and external lateral carinae of the pronotum about 1.4 mm wide. Posterior angles of the lateral lobes of the paranota extend obliquely, downwards and backwards, with rounded-truncated apices; ventral sinus present while tegminal sinus absent.

***Wings*.** Tegmina and hind wings reduced, very small, triangular, hidden beneath pronotum and invisible (the ‘abbreviated’ type after Zha et al. 2016).

***Legs*.** Dorsal and ventral margins of all femora finely serrate; fore and mid femora compressed, dorsal and ventral margins nearly straight; hind femur robust, about 2.6 times as long as wide, dorsal and ventral margins entire; antegenicular tooth slightly folded outwards with acute apex, apex of the genicular tooth obtuse; hind tibia with finely serrate inner margins, terminal part slightly wider than basal part, outer/inner side with 7–9/6–8 spines; first segment of hind tarsus 1.8 times as long as third, the first pulvillus short, while the second and third long, tips of all the pulvilli obtuse.

***Abdomen*.** Ovipositor narrow and long, upper valvae about 3.2 times as long as wide, outer margins of upper and lower valvae armed with saw-like teeth. Subgenital plate in ventral view: median carina entire and distinct; posterior margin truncated, in the middle has a broadly triangular protrusion which is folded inwards, base of the protrusion elevated and slightly higher than posterior margin.

***Coloration*.** Body dark brown. Antennae brown to dark brown. Pronotum behind shoulder usually has a pair of blackish spots (posthumeral spots), median carina of pronotum dotted with yellowish-brown. Ventral external area of hind femur mainly black, ventral margin of hind femur has a series of small yellow spots. Fore and mid tibiae with 3 yellowish-brown rings each, hind tibia with 2 elongate yellowish-brown rings.

##### Brief description of the males.

Slightly smaller than female. Antenna 15-segmented, with 8–10^th^ segments longest. The area between internal and external lateral carinae of the pronotum about 1.2–1.3 mm wide. Subgenital plate short and cone-shape, distal end nearly obliquely truncated in lateral view, apex bifurcate and forms into two short and obtuse teeth. Other characters same as females.

##### Measurements

(mm). Length of body♂10–11.5, ♀11–13.5; length of pronotum ♂7.5–8.0, ♀8.5–9.1; length of hind femur ♂6.4–6.9, ♀7.1–7.7, width of hind femur ♂2.5–2.7, ♀2.7–2.9; length of antenna ♂3.8–4.0, ♀4.1–4.5.

##### Type material.

***Holotype*** female, PR CHINA, Guizhou Province, Leishan County (Leigong-shan Mt.), 26°22'45.69"N, 108°11'42.83"E, 1460 m alt., 2 Aug. 2016, collected by Ling-Sheng Zha. ***Paratypes***: 15 males and 6 females, Leigong-shan Mt., 1400–1600 m alt., 1–3 Aug. 2016, collected by Ling-Sheng Zha.

##### Ecology and habits.

**Figure 3.**
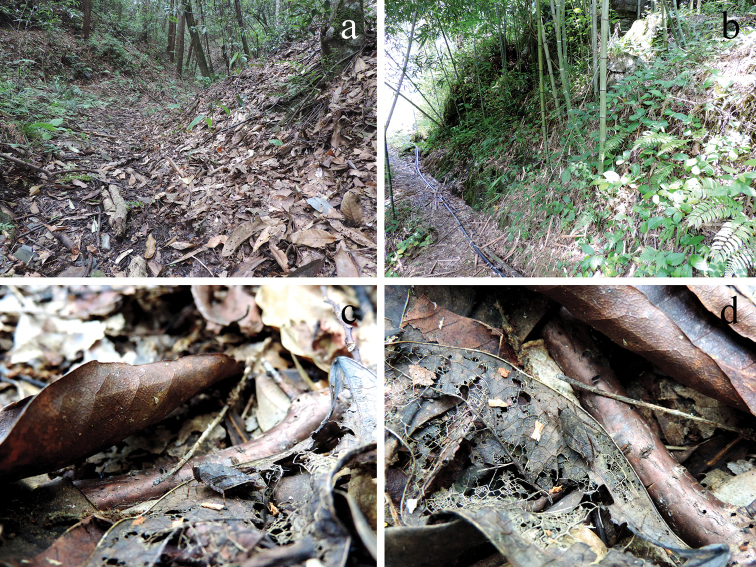
Habitats of two new *Formosatettix* species in PR China **a** habitat of *F. leigongshanensis* sp. nov. **b** habitat of *F. wulongensis* sp. nov. **c, d** female *F. leigongshanensis* sp. nov. standing on fall-leaf layers. Pictures **a, c, d** were photographed in Leigong-shan Mt., Leishan, Guizhou, China; while picture **b** was taken in Wulong, Chongqing, China.

Individuals of *Formosatettix leigongshanensis* sp. nov. inhabit fall-leaf layers in humid subtropical rainforests of Karst Region (Fig. 3a, c, d). They move slowly and can easily be caught. They mainly feed on humus. Specimens are capable of burrowing their bodies in shallow soil layer.

##### Etymology.

The new species is named after the type locality, Leigong-shan Mt., Leishan, Guizhou, China. The specific epithet is a third Latin declension adjective.

##### Distribution.

**Figure 4.**
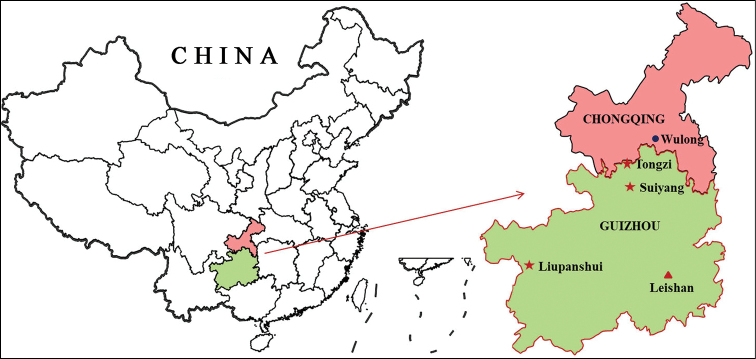
*Formosatettix* species inhabiting Guizhou and Chongqing, PR China. Red star: *F. serrifemora* from Liupanshui, Suiyang and Tongzi, Guizhou; Red triangle: *F. leigongshanensis* sp. nov. from Leishan, Guizhou; Blue circle: *F. wulongensis* sp. nov. from Wulong, Chongqing.

China (Guizhou). For now, only found in Leigong-shan Mt. in Leishan County (Fig. 4).

#### Formosatettix wulongensis

Taxon classificationAnimaliaOrthopteraTetrigidae

Zha & Ding sp. nov.

0E5BFB7D-8C59-5DC1-BB55-83E3A6DC898C

http://zoobank.org/A40391D1-ED6F-44DB-8317-1124F0A2F54B

Figs 5 , 6

##### Diagnosis.

*Formosatettix wulongensis* sp. nov. is similar to *F. serrifemora*, but the latter has elevated, arcuate and much more projected anterior margin of the vertex, narrower scutellum, lower arcuate median carina of pronotum in lateral view, and pointed-rounded apex of hind pronotal process (Wei et al. 2019, fig. 7b). Main differences between *F. serrifemora* and *F. wulongensis* sp. nov., together with *F. leigongshanensis* sp. nov., are outlined in Table 1.

*Formosatettix wulongensis* sp. nov. is also similar to *F. omeiensis* Zheng, 2009 from Sichuan and *F. baishuijiangensis* Zheng, 1999 from Gansu, China. *Formosatettix omeiensis* differs from our new species in 1) frontal costa together with the medial carina of the vertex acutely angled; 2) superior ocelli situated between the lower margin of the eyes; 3) anterior margin of the pronotum reaching only the level of the half length of the compound eyes; and 4) parallel prozonal carinae (Zheng 2009); while *F. baishuijiangensis* can be separated from the new species in 1) vertex 3.6 times as wide as one eye; 2) vertex strongly surpassing the anterior margin of the compound eyes; 3) scutellum 2 times as wide as the diameter of the scapus; and 4) anterior margin of the pronotum reaching only the posterior one-third of the compound eyes length (Zheng et al. 1999).

The new species is the first *Formosatettix* species known from Chongqing Autonomous Region, China.

**Figure 5.**
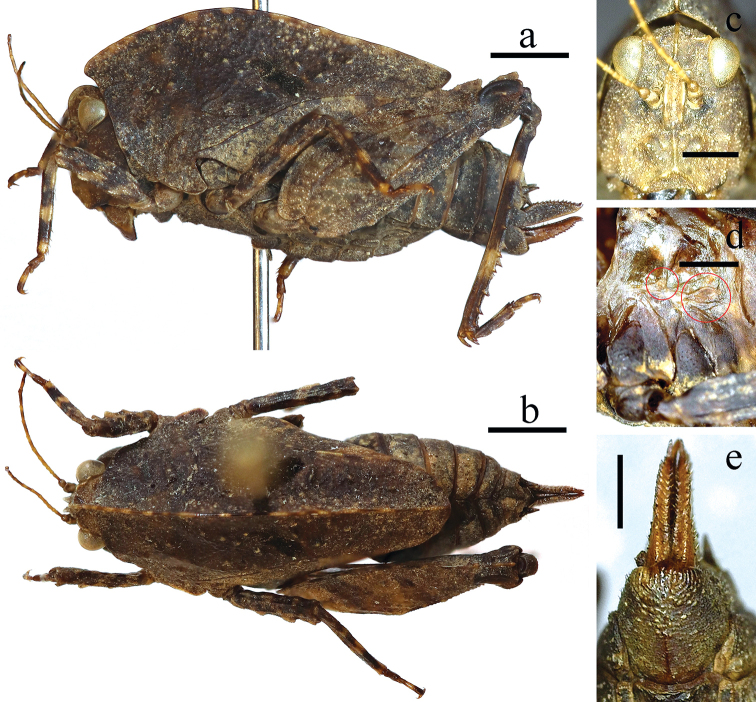
Female of *Formosatettix wulongensis* sp. nov. **a** body in lateral view **b** body in dorsal view **c** head in frontal view **d** reduced wings in lateral view (tegmenulum in the smaller circle, hind wing in the bigger circle) **e** subgenital plate in ventral view. Pictures **a, b** were stacked using Photoshop CS6. Scale bars: 2 mm (**a, b**), 1 mm (**c–e**).

**Figure 6.**
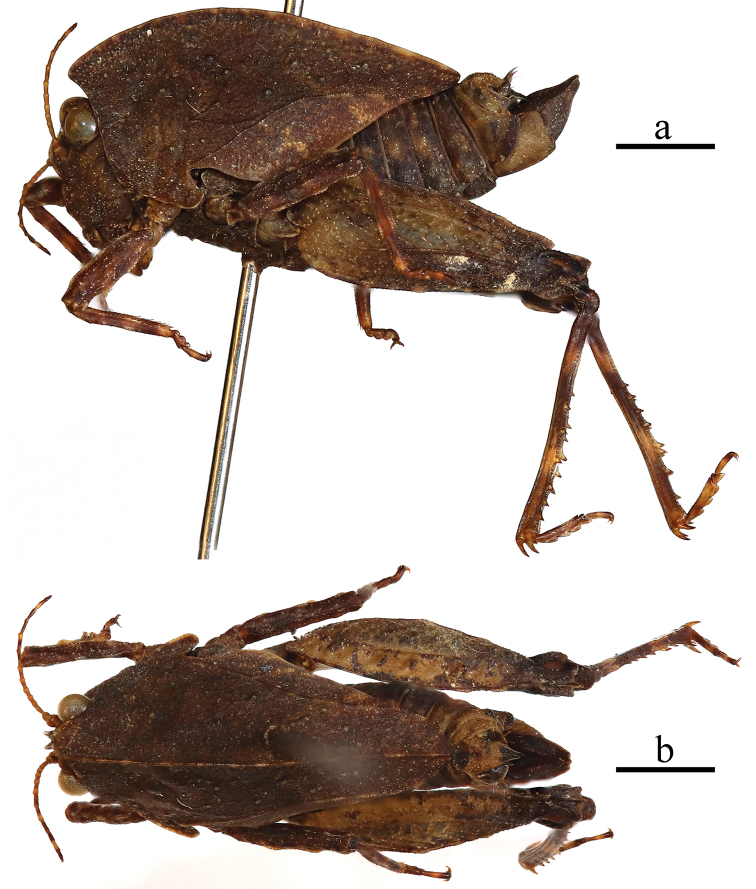
Male of *Formosatettix wulongensis* sp. nov. **a** body in lateral view **b** body in dorsal view. Pictures were stacked using Photoshop CS6. Scale bars: 2 mm.

##### Detailed description of female.

***General appearance*.** Body stout and short, size moderate; surface coarse, covered with numerous fine granules.

***Head*.** Vertex clearly below anterior margin of pronotum, 2.5 times as wide as a compound eye; middle portion higher than surrounding area; anterior margin straight and low, a little surpasses the anterior margin of the compound eyes; lateral carinae distinct, folded upwards and up to the top of eyes; medial carina distinct and compreso-elevated in the anterior half; paired fossulae distinct, rounded. In lateral view face nearly vertical; frontal costa together with medial carina rounded; facial carinae above superior ocelli distinctly concave, between the antennal grooves nearly obtusely triangular (strongly arcuate) forwards. In frontal view frontal costa bifurcates into facial carinae at the lower one-third of between anterior margin of vertex and upper margin of superior ocelli, and run nearly parallel downwards; scutellum deep and wide, between grooves 1.2–1.3 times as wide as the diameter of the scapus. Eyes globose and elevated over the anterior margin of pronotum, but clearly lower than vertex; superior ocelli placed slightly above the lower margin of eyes. ***Antenna*.** Antenna filiform, 16-segmented, inserted distinctly below the lower margin of eyes, segments 10–12^th^ longest and 4–5 times as long as wide.

***Pronotum*.** Pronotum strongly compresso-elevated, surface coarse, bearing small tubercles and wrinkles; between sulci a little swollen at the base of median carina and a little concave on both sides of the discus. Anterior margin projected forwards and nearly reaches the level of the anterior margin of eyes, in dorsal view acutely angled; prozonal carinae distinct, but short, slightly contracted backwards; hind pronotal process short, reaching 3/4 of the hind femur; apex broadly arcuate in dorsal view. Median carina of pronotum strongly compresso-elevated, in lateral view highly arcuate; lower margin of hind pronotal process curved, interlal lateral carinae incurved, the area between internal and external lateral carinae of the pronotum about 1.4 mm wide. Posterior angles of the lateral lobes of paranota directed downwards and backwards, with truncated or nearly truncated apices; ventral sinus present, tegminal sinus absent.

***Wings*.** Tegmina and hind wings extremely degenerated and very small, scaly, hidden beneath pronotum and invisible (the ‘abbreviated’ type after Zha et al. 2016).

***Legs*.** Dorsal and ventral margins of all the femora finely serrated; fore and mid femora compressed, dorsal margins almost straight, ventral margins with 3 teeth each (at the base, in the middle and at the end). Hind femur robust, about 2.8 times as long as wide, dorsal margin before antegenicular tooth bearing three lappets; ventral margins with a series of small teeth, three to four visible; ventro-external carina bearing a series of small teeth, 2–3 larger and evident; antegenicular tooth slightly folded outwards with apex acute, apex of genicular tooth obtuse; hind tibia with finely serrate inner margins, terminal part slightly wider than basal part, outer and inner sides with 5–7 spines each; first segment of hind tarsus 1.8 times as long as third, three pulvilli nearly equal in length and with obtuse apices.

***Abdomen*.** Ovipositor narrow and long; upper valvae about 3.2 times as long as wide; outer margins of upper and lower valvae armed with slender, saw-like teeth. Subgenital plate in ventral view: median carina evident in the anterior half while obscure in the posterior part; posterior margin truncated, in the middle with a broad triangular protrusion which is folded inwards.

***Coloration*.** Body dark brown. Antennae brown to dark brown from base to distal end, except for the pale colored 14^th^ segment; pronotum behind shoulder in some specimen has a pair of blackish spots (posthumeral spots); anterior margin, median carina and lateral carinae of pronotum covered in yellowish-brown dots; teeth on femora generally yellowish-brown; fore and mid tibiae with three yellowish-brown rings each; hind tibia with two elongate yellowish-brown rings.

##### Brief description of males.

Slightly smaller than female. Vertex 2.3 times as wide as one eye; antennae 15-segmented, segments 9–11^th^ longest; 13^th^ segment light colored. The area between internal and external lateral carinae of the pronotum 1.3 mm wide. Subgenital plate short cone-shape, distal part abruptly narrowed, distal end obliquely truncated in lateral view, but apex bifurcates into two distinct and obtuse teeth. Other characters same as females.

##### Measurements

(mm). Length of body ♂10.8, ♀12–14; length of pronotum ♂7.5, ♀8.2–9.2; length of hind femur ♂5.9, ♀6.3–6.8, width of hind femur ♂2.2, ♀2.3–2.5; length of antenna ♂3.5, ♀3.8–4.

##### Type material.

***Holotype*** female, PR CHINA, Chongqing Autonomous Region, Wulong County, 29°20'32.27"N, 107°45'23.35"E, 470 m alt., 11 July 2016, collected by Ling-Sheng Zha. ***Paratypes***: 1 male and 2 females, same data as holotype.

##### Ecology and habits.

Individuals of *F. wulongensis* sp. nov. inhabit slopes of bamboo forest in humid subtropical rainforests (Fig. 3b). They move slowly on fall-leaf layers among bushes. They may feed on mosses and/or humus. Most of their life cycles, they maybe burry their bodies in shallow soil layer.

##### Etymology.

The new species is named after the type locality, Wulong, Chongqing, China. The specific epithet is a third Latin declension adjective.

##### Distribution.

China (Chongqing). For now, only found in Wulong County (Fig. 4).

## Discussion

There has been a lot of discussion on how to properly describe the tegmina and hind wings of *Formosatettix*. Tinkham (1937) and Zheng (2005, 2009) described them as ‘absent or degenerated’, while Wei et al. (2019) referred to ‘tegmina’ as absent, while hind wings as ‘absent or very short’. Authors in other continents have similar issues, such as North America (Hancock 1902) or Europe (Skejo et al. 2014), and have already proven that the flying organs of some Tetriginae species are degenerated, not absent. We have now also checked the flying organs of the *Formosatettix* specimens (ten species altogether). Specimens of *Formosatettix* indeed have tegmina and hind wings present, but degenerated, very small, triangular and scaly or long-ovate, hind wind being distinctly longer than tegmenulum (Figs 1d, 5d). *Formosatettix* thus, has reduced tegmina and hind wings, not absent, just as members of the genus *Nomotettix* (Hancock 1902) in North America or *Tetrix nodulosa* and *Tetrix transsylvanica* in Europe (Skejo et al. 2014). We now have reasons to doubt that tegmina and hind wings of many brachypterous species in Tetrigidae taxonomy do exist, in reduced fashion, as they originated from winged ancestors. According to the classification standard of the hind wings of pygmy grasshoppers suggested by Zha et al. (2016, 2017a), flying organs of the members of the genus *Formosatettix* should be assigned to the ‘abbreviated’ type (hind wings never reach middle of hind pronotal process, but are distinctly longer than tegmina).

**Figure 7.**
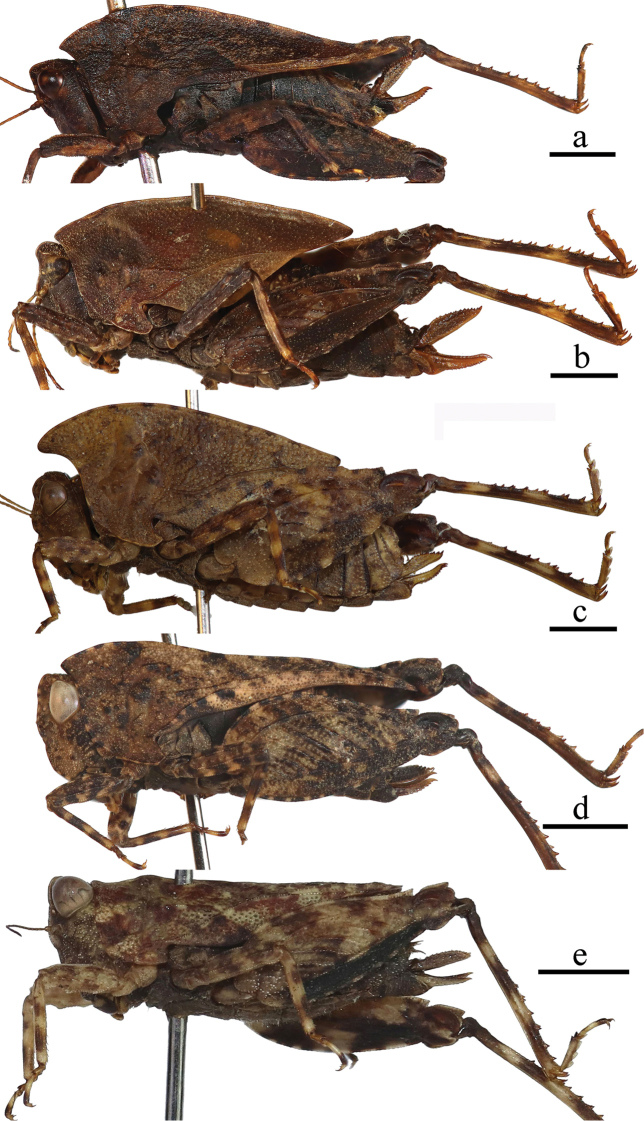
*Formosatettix* and its allies, comparison in lateral view **a***Alulatettix anhuiensis* Zheng **b***Formosatettix serrifemora***c***Deltonotus hainanensis* Zheng & Liang **d***Epitettix obtusus* Storozhenko & Dawwrueng **e***Macromotettixoides hainanensis* (Liang). Pictures were stacked using Photoshop CS6. Scale bars: 2 mm.

*Formosatettix* is morphologically similar to members of the genera *Alulatettix* and *Formosatettixoides* (Tetriginae), *Deltonotus* and *Epitettix* (with *Pseudepitettix* syn.) in (Cladonotinae), and *Macromotettixoides* (with *Pseudomacromotettix* syn.) (Metrodorinae). Based on previous work (Zheng 2005, Tumbrinck 2014, Zha et al. 2016, 2017a, b) and specimens in our hands, we provide brief comparison between *Formosatettix* and aforementioned genera, including summarized differences among Tetriginae, Cladonotinae and Metrodorinae (see Tables 2, 3; Fig. 7).

**Table 2.** Differences between Tetriginae, Cladonotinae and Metrodorinae (summarized based on Tumbrinck (2014) and our collections).

	Tetriginae	Cladonotinae	Metrodorinae
Medial carina of vertex in dorsal view	Reaching middle of vertex or more	Reaching one-third of vertex or less	Generally reaching 1/3–1/2 of vertex
Scutellum relationship to scapus	Narrower to slightly wider	In a few species slightly wider, in most species much wider	In some members narrower, in most slightly to clearly wider
Surface of pronotum	Generally smooth, humps absent, sometimes wrinkles present	Coarse, high or low humps present, sometimes wrinkles present	Relatively coarse, wrinkles generally present, humps absent
Direction of the lateral lobes of the paranota	Close to the body (downwards), sometimes indistinctly turned sidewards	From sidewards, to indistinctly or distinctly turned outwards	Directed sidewards or outwards

We furthermore suggest that the length of the medial carina of the vertex could be one of the important traits that could help in separating Cladonotinae from other related subfamilies (Table 2). *Alulatettix* differs from *Formosatettix* by clear tegminal sinus, usually absent or weak in *Formosatettix* (Table 3). Future research may discover numerous synonyms in Chinese Tetrigoidea, as already proposed in some studies. *Formosatettixoides* and *Formosatettix* could represent synonyms of each other, as well as *Epitettix* and *Pseudepitettix*, but also *Macromotettixoides* and *Pseudomacromotettix* (Table 3). Many *Formosatettix* species were either described as having wings absent, or lack photographs to be checked. Revisions are necessary in the future, as is good taxonomic practice (Lehmann et al. 2017).

**Table 3.** Differences between *Formosatettix* and its allied genera (together with the differences among the subfamilies outlined in Table 2, Fig. 7).

	***Alulatettix* (Tetriginae)**	***Formosatettix* and *Formosatettixoides* (Tetriginae)**	***Deltonotus* (Cladonotinae)**	***Epitettix* and *Pseudepitettix* (Cladonotinae)**	***Macromotettixoides* and *Pseudomacromotettix* (Metrodorinae)**
Tegminal sinus	Visible	Absent or weak	Absent	Absent or weak	Absent or weak
Tegmina and hind wings (uncovering pronotum needed!)*	Abbreviated	Abbreviated	Apterous	Vestigial (?)	Abbreviated
Anterior margin of the pronotum	Weakly elevated, not reaching the level of the anterior margin of the compound eyes	Weakly elevated, not reaching the level of the anterior margin of the compound eyes	Strongly elevated, in most species surpasses the level of the anterior margin of the compound eyes	Not at all or weakly elevated, not reaching the level of the anterior margin of the compound eyes	Not elevated, not reaching the level of the anterior margin of the compound eyes

* Hind wings of Tetrigidae can be divided into four types: ‘normal’ (developed, surpassing middle of hind pronotal process), ‘abbreviated’ (never reaching middle of hind pronotal process, but distinctly longer than tegmina), ‘vestigial’ (equal to or shorter than tegmina) and ‘apterous’ (completely absent) (Zha et al. 2016, 2017a). *Deltonotus* is considered as the ‘apterous’ type based on Storozhenko (2011) and confirmed by us on *Deltonotus hainanensis* Zheng & Liang; *Epitettix*, as well as *Yunnantettix* Zheng (Zha et al. 2016), is probably the ‘vestigial’ type, observed on *Epitettix obtusus* Storozhenko & Dawwrueng.

## Supplementary Material

XML Treatment for Formosatettix leigongshanensis

XML Treatment for Formosatettix wulongensis
